# Enzyme-Based Biosensors: Tackling Electron Transfer Issues

**DOI:** 10.3390/s20123517

**Published:** 2020-06-21

**Authors:** Paolo Bollella, Evgeny Katz

**Affiliations:** Department of Chemistry and Biomolecular Science, Clarkson University, Potsdam, New York, NY 13699-5810, USA; ekatz@clarkson.edu

**Keywords:** enzyme-based biosensors, direct electron transfer (DET), redox enzymes, nanostructured electrodes, protein film voltammetry (PFV)

## Abstract

This review summarizes the fundamentals of the phenomenon of electron transfer (ET) reactions occurring in redox enzymes that were widely employed for the development of electroanalytical devices, like biosensors, and enzymatic fuel cells (EFCs). A brief introduction on the ET observed in proteins/enzymes and its paradigms (e.g., classification of ET mechanisms, maximal distance at which is observed direct electron transfer, etc.) are given. Moreover, the theoretical aspects related to direct electron transfer (DET) are resumed as a guideline for newcomers to the field. Snapshots on the ET theory formulated by Rudolph A. Marcus and on the mathematical model used to calculate the ET rate constant formulated by Laviron are provided. Particular attention is devoted to the case of glucose oxidase (GOx) that has been erroneously classified as an enzyme able to transfer electrons directly. Thereafter, all tools available to investigate ET issues are reported addressing the discussions toward the development of new methodology to tackle ET issues. In conclusion, the trends toward upcoming practical applications are suggested as well as some directions in fundamental studies of bioelectrochemistry.

## 1. Introduction

**Figure 1:**
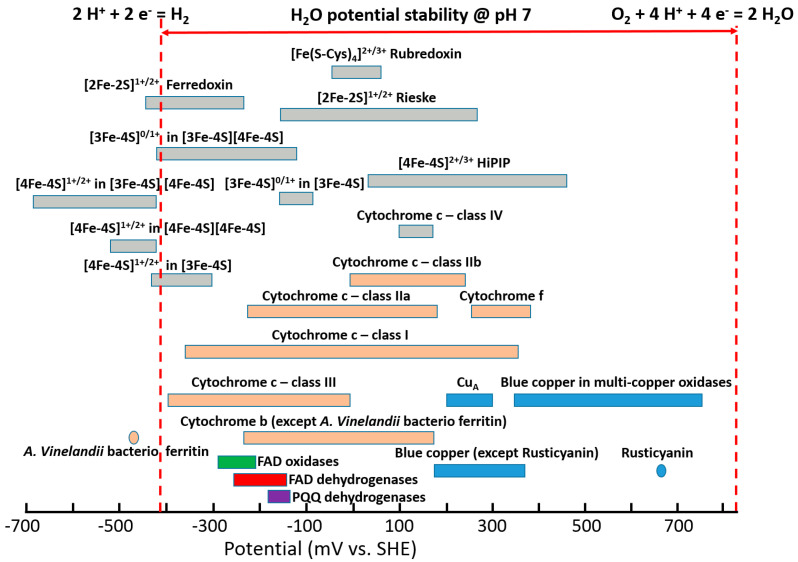
Standard potentials (E°) of various redox proteins and enzymes measured at pH 7.0 and recalculated vs. standard hydrogen electrode (SHE) reference. The potentials spread over range of values for the species originating from different biological sources. The data (except FAD oxidases/dehydrogenases and PQQ dehydrogenases) is adopted from [3] with permission. The potentials of FAD oxidases originate from refs. [7,8]; the potentials of FAD dehydrogenases originate from refs. [9,10,11]; the potentials of PQQ dehydrogenases originate from [12,13,14,15].

Redox enzymes are defined as proteins that facilitate biological electron transfer (ET) processes, acquitting for multiple essential biological functions like photosynthesis, respiration, nucleic acid biosynthesis, etc. [1,2,3]. Redox cofactors within the enzymes exhibit different ET thermodynamics and kinetics [4,5,6]. Moreover, redox cofactors exhibit different formal potentials (E°) spread over a potential window of approximately 1.5 V [3,7,8,9,10,11,12,13,14,15], which is wider, especially compared to the water thermodynamic stability window, Figure 1, considering hydrogen ions reduction to molecular hydrogen (E^0^’_2H^+^/H2_ = −0.41 V vs. standard hydrogen electrode (SHE) at pH 7) and oxygen reduction to water (E^0^’_O2/H2O_ = +0.82 V vs. SHE at pH 7) normally occurring in biological systems [16,17]. Recently, it was demonstrated how the potential of redox cofactors is affected by the redox center architecture and the surrounding protein structure [18,19,20]. 

The driving force in the investigation of biological redox molecules is mainly related to understanding the biochemical reactions being molecular bases of life [21]. Redox enzymes are extensively employed in the production of biofuels (e.g., hydrogen, methane, cellulose breakdown, etc.) [22,23,24]. However, they have also been used to develop new biocatalysts to solve challenging synthetic problems, to capture atmospheric CO_2_ [25]. Despite the great achievements in synthetic biology and green energy production, redox enzymes, being able to convert biological stimuli into electronic signals, are widely exploited in the development of electrochemical biosensors [26,27,28]. Among different sensing applications, the most famous example is certainly about blood glucose sensing, which greatly improved the life of billions of people worldwide [29,30,31].

**Figure 2:**
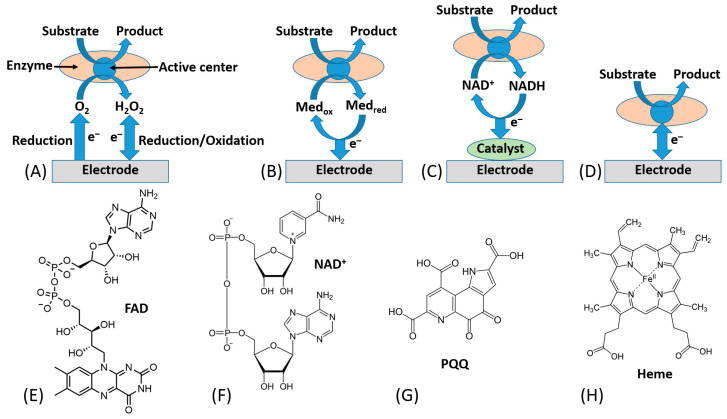
Different ways of electronic communication between redox enzymes and conductive electrodes: (**A**) Electrical communication through electrochemical transformations of enzyme substrate or product (exemplified with reduction of O_2_ and reduction/oxidation of H_2_O_2_ typical for oxidases). (**B**) Electrical communication using electron transfer mediators (relays) cyclic between oxidized (Med_ox_) and reduced (Med_red_) states (exemplified with an enzyme oxidizing a substrate and reducing a mediator, which is electrochemically re-oxidized and recycled back to Med_ox_). (**C**) Electrical communication using NAD^+^/NADH cofactor re-oxidized and recycled electrocatalytically (exemplified with an enzyme oxidizing a substrate and reducing NAD^+^ yielding NADH). (**D**) Electrical communication via direct electron transfer (DET) from an enzyme active center to an electrode (exemplified with an enzyme oxidizing a substrate and generating anodic current at an electrode). (**E**–**H**) Structures of the most typical enzyme redox cofactors: flavin adenine dinucleotide (FAD), nicotinamide adenine dinucleotide (NAD^+^), pyrroloquinoline quinone (PQQ) and heme.

In this research frame, most of bioelectrochemists have focused their attention on possible solutions to tackle direct electron transfer (DET) issues mainly for the development of sensitive, selective and stable biosensors [32,33,34]. The electronic coupling between redox enzymes and electrodes for the development of biosensors and biofuel cells can be accomplished according to three mechanisms, denoted as first-, second-, and third-generation biosensors. [35,36] Notably, first-generation biosensors are based on the electroactivity of a substrate or product of the enzymatic reaction [37] (Figure 2A). Second-generation biosensors based on mediated electron transfer (MET) use redox mediators (relays), which are small electroactive molecules shuttling electrons between the enzyme active sites and an electrode [38], Figure 2B. These can be freely diffusing mediators or bound to side chains of flexible redox polymers. In this class, we certainly include all enzymes that are using freely diffusing nicotinamide dinucleotide (NAD^+^) as primary electron acceptor, which later needs an immobilized catalyst (e.g., phenothiazines or quinones, particularly including pyrroloquinoline quinone (PQQ) [39], etc.) to reoxidize (recycle) NADH [40,41], Figure 2C. 

**Figure 3:**
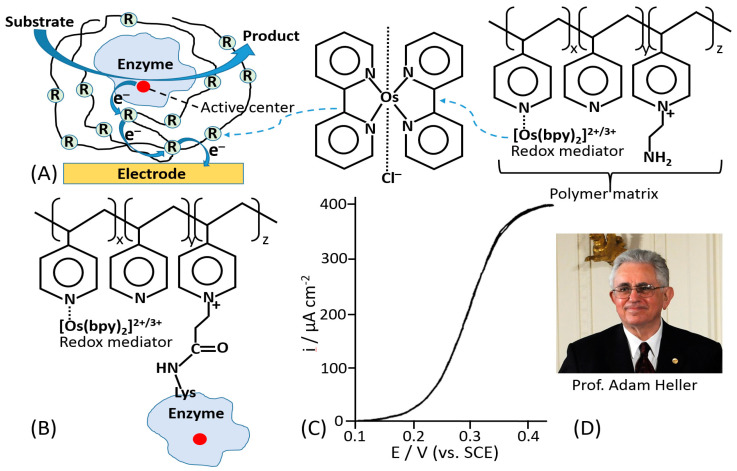
(**A**) An enzyme physically entrapped in a polymer operating as a redox matrix providing the enzyme electrical wiring by electron hopping through redox sites finally reaching an electrode conducting support. The redox-mediator sites are represented with [Os-(2,2′-bipyridine)_2_]^2+/3+^-complex pendant at poly(vinylpyridine) matrix. (**B**) A similar system where the enzyme is covalently bound to the redox polymer. The systems have been pioneered by Prof. Adam Heller. They are exemplified with an enzyme oxidizing a substrate and generating anodic current mediated by the redox polymer irrespective of the enzyme orientation. (**C**) A cyclic voltammogram, showing a 400 μA cm^−2^ glucose diffusion limited current density reached at 40 mM glucose concentration with the wired-enzyme shown schematically in (**B**). The scan rate is 5 mV/s. (**D**) Prof. Adam Heller – the pioneer in the enzyme wiring according to many various approaches, particularly including systems exemplified in (**A**,**B**). (Part C was adopted from [44]; (**D**) the photo was adopted from Wikipedia, public domain).

**Figure 4:**
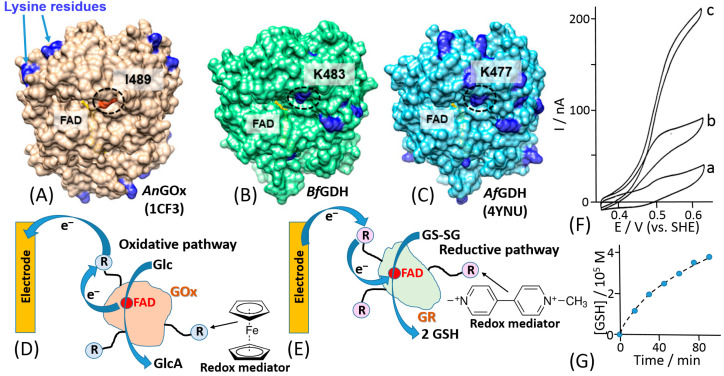
(**A**–**C**) Comparison of the positions of lysine residues in *Aspergillus niger* derived glucose oxidase (*An*GOx) (PDB ID: 1CF3), *Botryotinia fuckeliana* derived glucose dehydrogenase (*Bf*GDH) (model), and *A.flavus* derived GDH (*Af*GDH) (PDB ID: 4YNU). Lysine residues are shown in dark blue. In *Bf*GDH and *Af*GDH, lysine residues (K483, K477, circled) are located at the entrance of what appears to be a pathway to the active center. In *An*GOx, an isoleucine residue (I489, circled) is located at this position. (**D**) The electron transfer from soluble GOx to a Au electrode mediated by ferrocene redox relay (R) species covalently tethered to the enzyme with long flexible chains. Note that ferrocene has a positive redox potential needed to mediate the oxidative biocatalytic process. The biocatalytic reaction results in glucose (Glc) oxidation and gluconic acid (GlcA) formation. (**E**) The electron transfer from a Au electrode to soluble glutathione reductase (GR) mediated by viologen redox relay species covalently tethered to the enzyme with long flexible chains. Note that viologen has a very negative redox potential needed to mediate the reductive biocatalytic process. The biocatalytic reaction results in transformation of the oxidized glutathione (GS-SG) to the reduced glutathione (G-SH). (**F**) Cyclic voltammograms obtained with a bare (unmodified) Au electrode (a disk of 1.5 mm diameter) measured in the presence of a ferrocene-functionalized GOx (12 ferrocene electron relays per a GOx molecule, shown schematically in (**D**); 10 mg/mL): (a) in the absence of glucose; (b) and (c) in the presence of 0.8 mM and 5 mM glucose, respectively. A phosphate buffer solution (0.085 M, pH 7.0) was used as a background electrolyte applied under N_2_ atmosphere. Scan rate was 2 mV/s. (**G**) Formation of reduced glutathione bioelectrocatalyzed by GR functionalized with viologen mediator units tethered to the enzyme with long flexible chains (see experimental details in [48]. (Part A is adopted from ref. [46] with permission; part F is adopted from [20] with permission.)

The mediated ET has been achieved in systems of different complexity [1], ranging from very simple diffusion-operating soluble electron transfer mediators to very sophisticated molecular “machines” shuttling electrons between redox active centers of immobilized enzymes and a conductive electrode support [42]. A very efficient and at the same time simple construct was based on a redox enzyme (e.g., glucose oxidase, GOx) immobilized in a polymer matrix with pendant redox mediator units [43,44]. The enzyme was physically entrapped into the polymer matrix (Figure 3A) or covalently bound to the polymer chain (Figure 3B). The electrocatalytic (ET) current [44] (Figure 3C) has been achieved with random electron hopping from a mediator site to another site, finally reaching an electrode surface. This approach, pioneered by Adam Heller (Figure 3D), was one of the first effective electronic coupling of redox enzymes with electrodes. Another approach has been developed using redox groups tethered to an enzyme backbone, then operating as electron-transporting stations through quasi-diffusional conformational changes in the linker, if the linker was long enough to provide flexibility and mobility of the bound redox mediator [45]. Importantly, the location of the linker should be close to the catalytically active enzyme center. When amino groups of lysine residues are used for the covalent binding of the mediator, their position in different enzymes is important [46] (Figure 4A–C). The mediator-functionalized enzymes first operated in a solution [45] (Figure 4D,E) and then were immobilized at an electrode surface [47]. The tethered mediator facilitated ET from oxidizing enzymes to electrodes (providing anodic current) [20,45] (Figure 4D,F) or to reducing enzymes (providing cathodic flow of electrons) [48,49] (Figure 4E,G), depending on the type of the enzyme and appropriate redox potential of the bound mediator.

Third generation biosensors or DET-based biosensors are realized with the direct electronic connection between the redox center of the enzyme and the electrode surface, which is working as a signal transducer [50], Figure 2D. 

From the perspective of biosensing application, the third-generation electrode platform based on DET mechanism shows important advantages compared to MET, considering both soluble and immobilized mediator, and first generation. First of all, the absence of mediators and electroactive substrates/products allows a higher selectivity because the biosensor can operate at a potential closer to the E° of the redox enzyme, thus reducing possible interfering reactions. Second, both soluble/immobilized mediators and electroactive substrates/products may also facilitate unspecific reactions. Next, the absence of a reagent in the reaction sequence makes the device easier to realize. However, as mentioned above adsorbed/immobilized mediators allow the realization of reagentless biosensors (no freely diffusing mediator in solution), which is an obvious advantage compared to other second-generation biosensors that rely on the addition of mediators to sensing solution [2,34].

Today, an efficient ET connection between a variety of electrodes and a wide range of redox enzymes has been accomplished for many redox enzymes (e.g., flavin adenine dinucleotide (FAD), nicotinamide dinucleotide (NAD^+^), pyrroloquinoline quinone (PQQ), or heme-based redox enzymes) [51]. The chemical structures of their cofactors are shown in Figure 2E–H.

**Figure 5:**
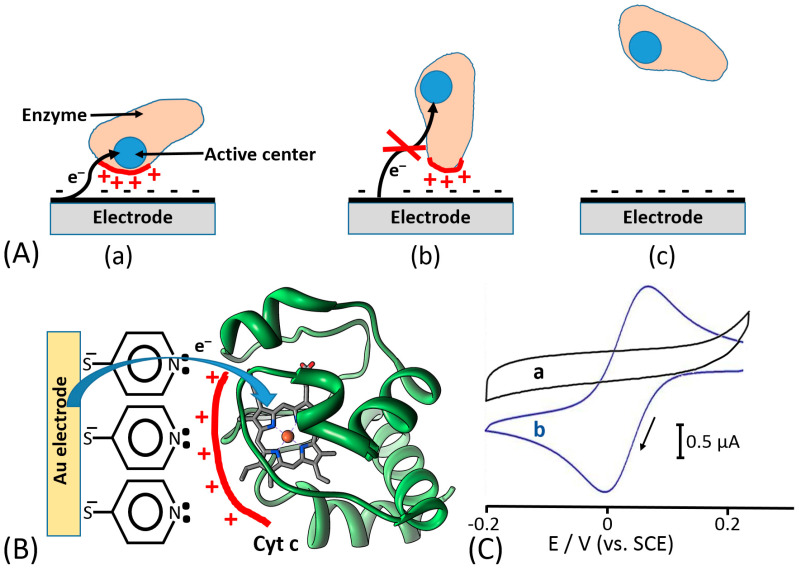
(**A**) Non-specific protein adsorption outcomes: (a) Electrostatic attraction of oppositely charged protein residues and electrode surface results in immobilization of the protein in an electroactive orientation, facilitating direct electron transfer between a redox center and the electrode. (b) Protein becomes adsorbed in an orientation that does not facilitate direct electron transfer. (c) Protein does not adsorb to the electrode surface and the direct electron transfer is not possible. (**B**) Alignment of cytochrome c (Cyt c) at a Au electrode surface functionalized with a promoter self-assembled monolayer. The alignment results in a short distance between the heme active center facilitating the direct electron transfer. (**C**) The cyclic voltammograms obtained in the presence of Cyt c (0.1 mM): (a) at a bare Au electrode without the protein alignment and with no direct electron transfer; (b) at a modified electrode (as shown in (B)) with the alignment facilitating the direct electron transfer. Potential scan rate is 50 mV s^−1^. Phosphate buffer (1 mM, pH 7.0) under Ar was used as a background electrolyte.

**Figure 6:**
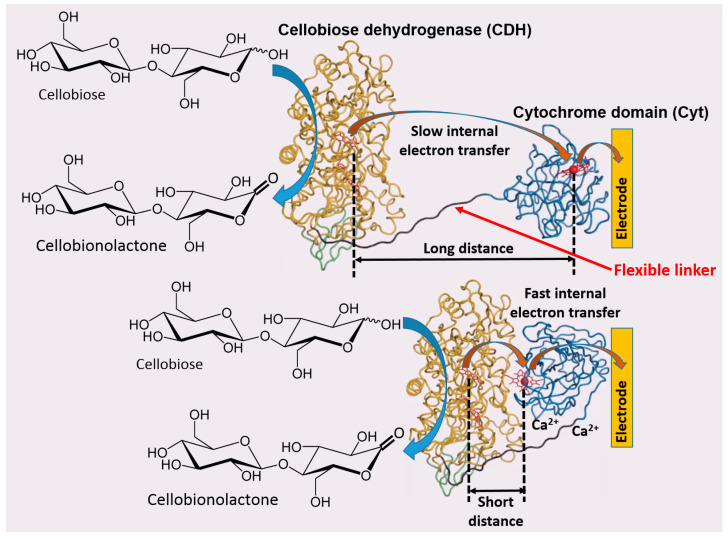
Direct electron transfer from the immobilized cellobiose dehydrogenase (CDH) catalytic domain to an electrode via a covalently linked cytochrome (Cyt) domain. The bioelectrocatalytic current depends on the conformation of the flexible linker. The short electron transfer path resulting in facilitation of the current was realized in the presence of Ca^2+^ cations.

Based on the previous literature, electrostatic compatibility between an electrode and protein surface (part of the surface responsible for ET) seems to play a key role in order to establish an efficient DET, thus showing fully reversible or quasi-reversible cyclic voltammograms in non-turnover conditions (in the absence of an enzyme substrate) [52,53,54]. Thereafter, the reversibility of non-turnover cyclic voltammograms (depending on the ET rate) will affect the catalytic current produced in the presence of an enzyme substrate. Moreover, the polarity of redox enzyme/electrode interfaces is dramatically affecting the enzyme molecules adsorption and orientation onto the electrode, thus sometimes not facilitating DET processes or even hindering their adsorption, which impedes any biological ET without relying on redox mediators [55,56] (Figure 5A). In this regard, another important aspect, that has been deeply investigated about the enzyme–enzyme and enzyme–interface interactions, is the ability of small multivalent cations to promote the ET between negatively charged proteins and electrodes (e.g., Mg^2+^ or Ca^2+^, which are ubiquitous in nature) [57]. In this regard, Schulz and his co-workers have been able to increase the catalytic activity of cellobiose dehydrogenase (CDH) by the addition of CaCl_2_ to the buffer [58,59] (Figure 6). Cellobiose dehydrogenase (CDH, EC 1.1.99.18) is an extracellular monomeric redox enzyme that consists of a catalytically active dehydrogenase domain (CDH_DH_), connected through a flexible linker region to a cytochrome domain (CDH_CYT_). During the catalytic process, carbohydrates (e.g., cellobiose, lactose or glucose) undergo two electrons oxidation at the FAD cofactor (CDH_DH_) subsequently transferring electrons from CDH_DH_ to CDH_CYT_ by an internal electron transfer (IET) process. The reduced CDH_CYT_ further transfers electrons to one-electron molecule acceptors, like cytochrome *c* (Cyt *c*), in a biological process or to a macroscopic electrode in a bioelectrochemical process [60,61,62]. The observed effects of especially divalent Ca^2+^ on the catalytic currents (increased up to five times) can be ascribed to a modified interaction between CDH_CYT_ and the electrode and/or between CDH_CYT_ and CDH_DH_. Regarding the IET, most probably Ca^2+^ ions are complexed by the carboxyl groups of aspartic and glutamic acid at the interface of the CDH_DH_ and CDH_CYT_ domains, thus resulting in a closer domain interaction and a higher IET rate. This concept has been recently demonstrated also for fructose dehydrogenase (FDH), which exhibits a similar structure compared to CDH [63]. 

Electrostatic interactions or hydrogen bond formation between redox proteins (e.g., cytochrome *c*) and monolayer-functionalized electrode surfaces have been used to align proteins at an electrode surface providing a short distance between the redox cofactor and conducting interface (Figure 5B), thus allowing reversible electron transfer [64,65], which was impossible without the orientation effect for the protein molecules (Figure 5C). 

In addition to the electrostatic forces that are affecting the enzyme orientation, DET efficiency is also affected by the internal electron tunneling distances. On this specific aspect, Harry Gray and his co-workers demonstrated that electron tunneling distances play a key role in the ET between electron-donor and electron-acceptor partner redox-active centers, thus affecting the ET rate [66,67,68]. According to Guo and Hill theory [69], the enzymes can be classified in intrinsic enzymes, in which there are no pathways for the electron tunneling because of the absence of appropriate redox sites, and extrinsic enzymes, in which there is a redox acceptor allowing the electron tunneling toward the electrode. Moreover, Dutton et al. established a simple and practical rule that within metalloenzyme structures, high ET rates are supported by an electron tunneling distance of less than 14 Å between redox active sites and electrodes avoiding limiting steps in the redox catalysis. Ideally, in order to allow efficient ET, all enzyme molecules would adsorb and orient on the electrode with the same sub-14 Å distance between the redox cofactor and the electrode [70].

This review aims at summarizing all findings about DET of redox enzymes with a special focus on theoretical (e.g., Marcus theory) and practical aspects (e.g., electrochemical techniques used to study DET and (bio)engineering approaches used to tackle DET issues). A particular attention will be devoted to the case of glucose oxidase (GOx, E.C. 1.1.3.4) from *Aspergillus niger* that has been widely and wrongly used to develop DET based biosensors. Despite the huge number of publications on this subject, that unfortunately accounts for thousands of citations, there is no solid evidence to support DET in GOx based on a stunning statement made by George Wilson: “based on recent experimental results, the observed electrochemical signal corresponds to the FAD cofactor non-covalently bound to the enzyme scaffold that comes out from the redox enzyme upon application of potential, getting adsorbed onto the electrode surface” [71].

## 2. Theoretical Aspects of Electron Transfer (ET) Processes

### 2.1. Marcus Theory

**Figure 7:**
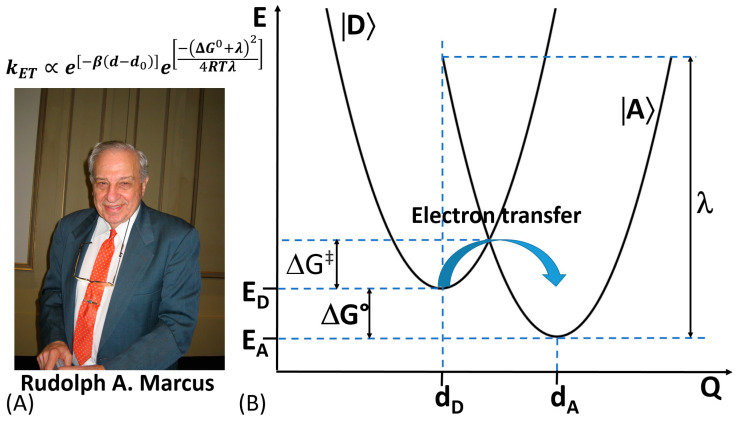
(**A**) Rudolph A. Marcus, the Nobel Prize in Chemistry (1992) recipient “for his contributions to the theory of electron transfer reactions in chemical systems”. (**B**) Profile of potential energy (E) surfaces vs. nuclear coordinates (Q) for a reactant, an electron donor (|D〉), and a product, electron acceptor (|A〉), corresponding to an electron transfer reaction: d_D_ and d_A_—coordinates corresponding to the energy minimum of the reactant and product, respectively; E_D_ and E_A_ minimum energies (redox potentials) of the reactant and product, respectively; ΔG° and ΔG^‡^—free Gibbs energy change and activation energy, respectively, in the course of the electron transfer reaction; λ—reorganization energy upon transition from the reactant to the product. The equation is the theoretical expression derived by R. A. Marcus for the electron transfer reaction rate dependence on the energy parameters and electron transfer distance (see explanations for all parameters in [75]. (The photo is adopted from Wikipedia, public domain.)

In 1992, Rudolph A. Marcus was awarded with the Nobel Prize in Chemistry for his contribution to the development of the ET theory in chemical systems [72,73] (Figure 7A). The theory takes into consideration changes in the structure of the reacting molecules and the solvent’s molecules. Based on changes in the energy of the molecular systems, the rate of chemical reactions can be calculated [74].

In an ET reaction, we must first define the electron donor species (D) and the electron accepting ones (A). To enhance the coupling probability of their electronic orbitals, D and A (reactants) should be as close as possible. On the other hand, both vibrational and orientational (affected by surrounding environment) coordinates are varying around the equilibrium values mainly due to charge transfer occurring during the ET process [75]. The potential energy of D and A as reactants and products is expressed as a function of their nuclear coordinates, which can be represented in a multidimensional potential-energy surface (Figure 7B). It should be emphasized that biological ET shares certain features in common with the ET in chemical systems [76]. However, despite the similarities in the ET in chemical and biological systems, we should also consider some substantial differences typical for biochemical reactions—(*i*) lack of self-exchange reactions, (*ii*) slightly less available structural information compared to chemical systems, (*iii*) less homogeneous environment for the ET in biological systems, (*iv*) lack of free energy (ΔG^0^) data for biological systems, (*v*) ΔG^0^ data are dependent on electric potential across the membrane of biological systems, (*vi*) protein conformational changes may precede or follow the electron transfer in biological systems, thus the binding free energy might differ between the oxidized and reduced form of redox cofactor, and (*vii*) hindering the possibility of any contacts between donator and acceptor redox center due to physical constrains (e.g., redox centers should have locked position in biological systems) [77]. 

In biological systems, the dependence of the ET rate on the distance between D and A has been widely elucidated both theoretically and experimentally. In this regard, the ET theory for biological systems, named afterwards “Marcus Theory”, is able to predict the ET rate constant value as given by Equation (1):

(1) $$k_{ET}\propto e^{\left[-\beta \left(d-d_{0}\right)\right]}e^{\left[\frac{-{\left({\Delta G}^{0}+\lambda \right)}^{2}}{4\mathrm{RT}\lambda }\right]}$$

where *β* corresponds to the decay or attenuation factor (about 10 nm^−1^ for proteins), ΔG^0^ and λ correspond to the free Gibbs energy and reorganization energy accompanying the ET process; d_0_ and d are the Van der Waals distance and actual distance between redox active sites; while R and T have their usual meanings [78].

Considering biological D and A reactants similar to those involved in a long range non-biological intramolecular ET, the ET rate constant can be approximated as e^−βr^, so exponentially dependent on the distance (β_r_) between D and A (as reactants). In the aforementioned formula, the ET rate constant is also dependent on intrinsic (λ) and thermodynamic (ΔG^0^) factors as well as dependent from the mutual orientation of the reactants. Although this is the universal rule to study and improve DET connection between redox enzymes and electrodes, other formulas and models have been proposed to calculate the ET rate at an electrode surface.

### 2.2. Other Theoretical Aspects

Today, many bioelectrochemists are using the model proposed by Laviron to compute the ET rate constant valid for diffusionless (surface-confined) electrochemical systems. The model was derived at first considering linear sweep voltammetry measurements, and it can be applied for any degree of reversibility of the electrochemical reactions [79,80]. However, the main constrain of the model is that both the oxidized and reduced forms of redox species should be strongly adsorbed (immobilized) onto the electrode surface. The first theoretical approximation was derived with the assumption that the adsorbed species do not interact with each other (Langmuir isotherm) [79]. The ET rate constant can be calculated considering the trend between the variation of peak potentials (both anodic and cathodic) toward the logarithm of potential scan rates. The heterogeneous electron transfer rate constant (k_s_) for adsorbed (and eventually monolayer immobilized) species can be calculated as follows (Equation (2)):

(2) $$\log k_{s}=\alpha \log\left(1-\alpha \right)+\left(1-\alpha \right)\log\alpha -\log\left(\frac{RT}{nF\nu }\right)-\alpha \left(1-\alpha \right)nF\frac{\Delta E_{p}}{2.3RT}$$

where *α* denotes the electron transfer coefficient, *k_s_* is the standard rate constant of the surface reaction, *ν* represents the potential scan rate, *n* is the number of electrons transferred, *RT* is the gas constant and absolute temperature (K), and Δ*E_p_* is the peak-to-peak separation. The experimentally determined Δ*E_p_* (the difference between the anodic and cathodic peaks; Δ*E_p_* = *E_pa_* − *E_pc_*) can be found from linear sweep or cyclic voltammetry experiments. The calculated electron transfer rate constant, *k_s_*, can be only a rough estimated value because of many assumptions (mostly assuming Langmuir isotherm for the adsorbed redox species) used in the first theoretical approximation [79]. The second approximation developed by Laviron and Roullier [80] partially solved this problem taking into account possible interactions (attractive or repulsing) of the redox species in the monolayer. However, this theoretical treatment included many parameters which are usually unknown and difficult to find experimentally. Thus, the second Laviron’s approximation was rather useless for practical calculations. Overall, the experimental procedure required for the Laviron’s estimation of the rate constant corresponding to the interfacial electron transfer usually includes cyclic voltammetry performed with different potential scan rates, then finding Δ*E_p_* as a function of the logarithm of the potential scan rate. All other parameters can be found from independent experiments, thus allowing use of the Laviron’s equation (Equation (2)). 

## 3. Why Glucose Oxidase (GOx) Cannot Undergo DET?

In 1962, Clark and Lyons reported for the first time the employment of glucose oxidase (GOx) from *Aspergillus niger* (E.C. 1.1.3.4) for the development of an enzyme-based electrode [81]. This report has always been recognized by the scientific community as the year of birth of biosensors. Afterward, GOx was widely studied as a redox enzyme for many bioelectrochemical applications (e.g., biosensors, enzymatic fuel cells (EFCs), etc.) [31,82,83]. There are several reasons for its popularity [84]. It is commercially available at relatively cheap costs. Moreover, it is highly active, very stable, and robust as an enzyme [85]. However, the most important reason is its ability to oxidize *β*-D(+)-glucose, thus allowing monitoring of *β*-D(+)-glucose for clinical applications like the management of diabetes [29,86,87].

Since Clark’s initial paper, an enormous number of papers on electrochemical glucose biosensors have been published [88,89,90,91,92,93]. As earlier reported in this review, also amperometric glucose biosensors can be divided into three classes based on the type of ET mechanism. While the pioneering work of Clark was highly important for development of electrochemical biosensors, much more practically important results have been obtained with the second-generation biosensors based on mediated electron transfer (MET), greatly contributed by Anthony Cass and Adam Heller [43,44,94] who used ferrocene mediating electron transfer from GOx and included enzymes in polymeric redox matrices, respectively [43,44,94,95]. These works are certainly the most significant advancement in the topic of biosensors [96]. About the “third generation” glucose biosensors, there has always been an open debate on the possibility for GOx to transfer electrons directly from its FAD-cofactor to an electrode surface [71,97]. In this regard, Bartlett et al. recently managed to prove experimentally that there is no evidence to support DET of GOx, thus the vast majority of publications in the literature about DET of GOx are claiming misleading results [98]. This paper raised considerable attention of the scientific community, especially after a “strong” statement made by George Wilson (published as editorial of *Biosensors and Bioelectronics* in 2016 [71]), and today, it has been acknowledged and endorsed by all bioelectrochemists.

The main explanation is based on a deep and comprehensive analysis of the GOx enzymatic structure. The enzyme is a homodimer (composed of two identical units containing flavin adenine dinucleotide (FAD) active sites) highly glycosylated with a molecular weight (MW) of about 160 kDa (the MW is dependent on the level of glycosylation) [99]. The GOx enzyme exhibits high specificity for oxidation of β-anomer of D(+)-glucose and the reaction occurs through a “ping-pong” mechanism, where one of the oxidized FAD in the homodimer reacts with the substrate to give the reduced flavin (FADH_2_) and the product gluconolactone (which undergoes a subsequent hydrolysis in neutral solution to gluconic acid) [100], as shown in Equations (3a) and (3b).

β-D(+)-glucose + GOx(FAD) → D(+)-glucono-1,5-lactone + GOx(FADH_2_) (3a)

D(+)-glucono-1,5-lactone + H_2_O → gluconic acid (3b)

when the GOx-catalyzed reaction proceeds in an artificial system (not in a native biological environment) the reduced active centers, GOx(FADH_2_), in the homodimers are then oxidized by reaction with oxygen, regenerating the initial oxidized form GOx(FAD) and producing H_2_O_2_ as a byproduct (Equation (4)).

GOx(FADH_2_) + O_2_ → GOx(FAD) + H_2_O_2_ (4)

**Figure 8:**
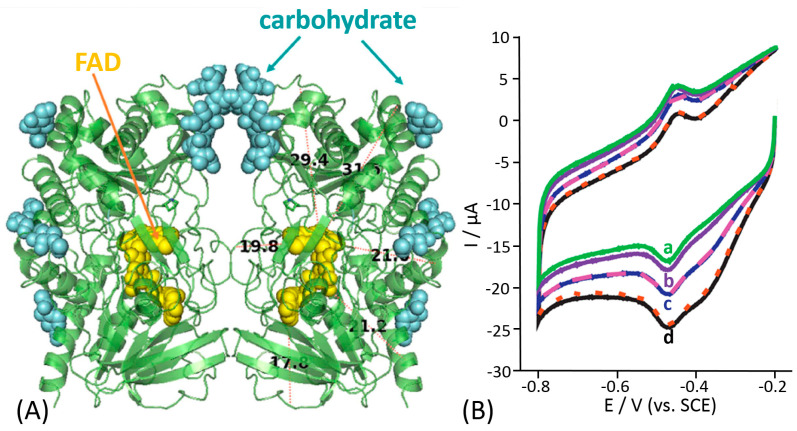
(**A**) Representation of the secondary structure of GOx. The image was obtained with PyMol software, PyMol visualizations are based on the crystal structure of GOx from *Aspergillus niger*, PDB code 1gal http://www.rcsb.org/pdb/explore/explore.do?structureId=1GAL. (**B**) Cyclic voltammograms showing oxygen reduction on a glassy carbon electrode modified with multi-walled carbon nanotubes and loaded with GOx: (a) in the absence of glucose, and in the presence of (b) 2 mM, (c) 4 mM and (d) 8 mM glucose. The experiment was performed in 0.1 M phosphate buffer, pH 6.8, in the presence of oxygen (in equilibrium with air) with the potential scan rate of 60 mV s^−1^. Note that this and similar cyclic voltammograms were erroneously reported as the proof of the DET with GOx. (The figure is adopted from ref. [98] with permission.)

Unfortunately, the majority of the early studies reported on glucose biosensors were performed without any knowledge of the enzyme crystal structure, thus without the knowledge of the distance separating the FAD cofactor and an electrode surface. Notably, the 3D structure of GOx was published by Hecht in 1993 [101,102,103] (Figure 8A). Based on the analysis of the GOx crystal structure, the two flavin active sites are deeply buried within the enzyme body, thus hindering any redox communication between the two dimeric units of the enzyme. From the crystal structure, it is possible to observe a distance of about 17–22 Å between the active sites and the enzyme surface. Actually, this distance was estimated for a deglycosylated enzyme; thus, in reality, distances might be larger considering that the molecular weight and size of the “native” (glycosylated) GOx are higher by 16-25% compared to the deglycosylated species [104,105,106]. Therefore, the DET for GOx is rather unlikely. Moreover, it is possible to observe the presence of a channel at the interface between the GOx homodimers. This structure actually hinders any possibility of non-specific electron transfer to electron acceptors available in biological systems and controls the local environment around the FAD cofactor ensuring high selectivity, not oxidizing any closely related carbohydrates. The turnover rate for the β-anomer of D(+)-glucose is 150 times higher than that for the α-anomer [107]. 

In most previous papers, GOx has been immobilized on carbon-based nanomaterials (e.g., carbon nanotubes, graphene, graphite, etc.) or metal-based nanomaterials (e.g., gold nanoparticles, porous gold, etc.) [108,109,110,111,112,113,114]. Concerning carbon nanomaterials, the claimed DET of GOx is often attributed to some “special” but not clearly specified, properties of the carbon nanomaterials or possibly some particular interactions of the enzyme and the carbon nanotubes that allow to access the active site, thus enabling the charge transfer between the FAD cofactor and carbon nanotubes [115,116,117,118].

In a typical cyclic voltammogram, the data reported to support these claims of DET for glucose oxidase are (*i*) a pair of peaks with the shape characteristic of surface-confined redox species at around −0.26 V vs. SHE at pH 7, assumed to be the FAD in the active enzyme center, and (*ii*) the catalytic current upon addition of glucose that correlates with the glucose concentration. Figure 8B shows the typical results usually considered as evidence for DET of GOx, without taking into account the real scientific meaning of the results. First, the current response shows a reductive “catalytic” current that cannot be considered as oxidation response expected from the enzymatically catalyzed oxidation of glucose. Second, the surface redox peaks around −0.26 V vs. SHE are unchanged by addition of D(+)-glucose with both oxidation and reduction peaks clearly present throughout and simply moving with the changing background. Third, the change in the current upon glucose addition starts at approximately 0 V vs. SHE, which does not match with the thermodynamic potential of FAD. To analyze and understand the results reported in Figure 8B, we should consider the fact that oxygen is dissolved in the solution. Most likely, the reductive “catalytic” wave correlates with the reduction of oxygen on carbon nanotubes electrode that starts at 0 V vs. SHE. The reductive “catalytic” current decreases because oxygen is serving as electron acceptor for the reaction catalyzed by the enzyme, thus being less available for its reduction at the electrode surface upon addition of different concentration of glucose [119]. In other words, the observed effect of glucose originates not from the glucose oxidation but rather from O_2_ depletion. Next, we should also consider that the reduction of oxygen produces mainly H_2_O_2_ in that potential window [120]. Moreover, H_2_O_2_ exhibits a large overpotential window on carbon nanotubes being thus unavailable for its further reduction to water. However, the reaction can be easily catalyzed in the presence of metal nanoparticles incorporated into carbon nanotubes on the electrode surface, therefore consuming the H_2_O_2_ produced from the enzymatic reaction [121,122,123]. It should be noted that the redox peaks observed (E^0^’= −0.26 V vs. SHE in pH 6.8) in non-turnover conditions cannot be taken as the evidence for the DET from the flavin in the active site of GOx to the electrode surface. Conversely, they most likely arise from free FAD adsorbed directly on the electrode surface. The free FAD can either be present as impurity of the enzyme sample or dissociate from the enzyme during the incubation of the electrode with the enzyme [98]. These experiments unequivocally prove that GOx immobilized on an electrode surface cannot undergo DET reaction mechanism. Unfortunately, many of these claims were usually supported by the sentence “the data presented are similar to previously reported literature”, without considering the reliability and the “correct” scientific meaning or interpretation of the results.

## 4. Methods to Investigate DET Issues

For bioelectrochemists, one of the most intriguing and recent challenges has been the construction of an electrode platform based on the DET mechanisms. Notably, to realize the DET mechanism and then to prove it many methods have been developed. Herein, we sort them in two classes: biochemical methods facilitating the DET (e.g., deglycosylation, enzyme mutation, etc.) and electrochemical methods investigating the process and providing the prove of the DET (e.g., cyclic voltammetry, amperometry, protein film voltammetry, etc.) [16,97,124,125]. 

### 4.1. Biochemical Methods

This section is resuming the main biochemical methods that have been widely employed to distinguish DET bioelectrocatalysis from other catalytic signals, originating possibly from a dissociated cofactor, like in the case with GOx. At first, we should take into account that small conformational changes in the enzyme may occur upon its immobilization at an electrode, thus, lowering also the enzyme activity. From the biochemical point of view, one control experiment cannot confirm the DET mechanism of the enzyme, thus multiple experiments are indeed required to prove the ET pathway of the enzyme immobilized on the electrode and to support the conclusion on the DET. 

For example, as the first experiment, we might consider determining the formation of the expected product, which does not give any confirmation for the ET mechanism, but it only tells that the reaction is actually taking place (it might occur at a dissociated cofactor or other reasons not related to the enzyme activity). Recently, Duca et al. co-immobilized nitrogenase with a noble metal catalyst in order to perform the stepwise reduction of nitrate to ammonia through its intermediate, namely nitrite [126]. Initially, nitrate reduction to nitrite was assayed by using the well-established Griess method [127] to detect nitrite, also confirmed by computing the theoretical nitrite amount produce during the bioelectrocatalytic process (the charge passed through the electrode during the catalytic process was correlated with the concentration of nitrite by using Coulomb’s law). After proving the intermediate, the authors used a fluorescent compound, namely (*o*-phthalaldehyde), to quantify the ammonia produced during the further reaction step catalyzed by the noble metal catalyst. This method is particularly useful when the whole biocatalytic process proceeds through several intermediate steps [128,129]. 

Besides the product analysis, we should consider the importance of enzyme tertiary and quaternary structure for its activity. Recently, it has been proved that estimating the activity loss occurring upon denaturation processes (e.g., heating to elevated temperature from 80 °C to 100 °C for a short time or by treating the enzyme with proteases, like trypsin, etc.), it is possible to differentiate between a DET bioelectroctalytic mechanism and a cofactor that dissociates from the enzyme and undergo only electrocatalysis (without any contribution from the enzymatic structure to selectivity and turnover) [130,131]. Additionally, if the enzyme exhibits a complex ET mechanism it is possible to add different inhibitors during bioelectrocatalytic measurements, thus determining all the steps and ET mechanisms contributing to the enzyme turnover [132,133,134]. 

Although previously reported methods are easy to perform, one of the most appealing method to confirm possible DET still remains the generation of mutated or modified oxidoreductases that exhibit altered catalytic properties, sometimes depending on the orientation and immobilization at the electrode surface [135]. These mutations certainly include single-point mutations, the cleavage of component subunits or even the deglycosylation of enzymes [136]. Consequently, these might alter the apparent kinetics parameters like maximum reaction rate, *V*_MAX_, or the Michaelis constant, K_M_. Mutations can also induce modification of the ET mechanism or internal electron-tunneling pathway. For example, Léger et al., who altered the ligation of the distal [4Fe–4S] cluster in a hydrogenase, by replacing a histidine residue with a glycine residue. The DET bioelectrocatalysis was significantly facilitated as the result of this change in the protein backbone [137]. However, the generation of mutant enzymes is neither straightforward nor trivial in the majority of the cases. 

Finally, the potential applied to the electrode may indicate whether direct bioelectrocatalysis of active enzyme is occurring. This assumption is based on the fact that the reduction potential of the enzyme’s cofactor has been predetermined and that its reduction potential is not mildly altered upon any small conformational changes that may occur by the enzyme immobilization.

### 4.2. Electrochemical Methods

Classical electrochemical methods like cyclic voltammetry, linear sweep voltammetry, differential pulse voltammetry and chronoamperometry have been widely employed to investigate the catalytic properties of many enzyme-modified electrodes giving important insight on their apparent kinetics properties and also on the ET mechanism [138].

**Figure 9:**
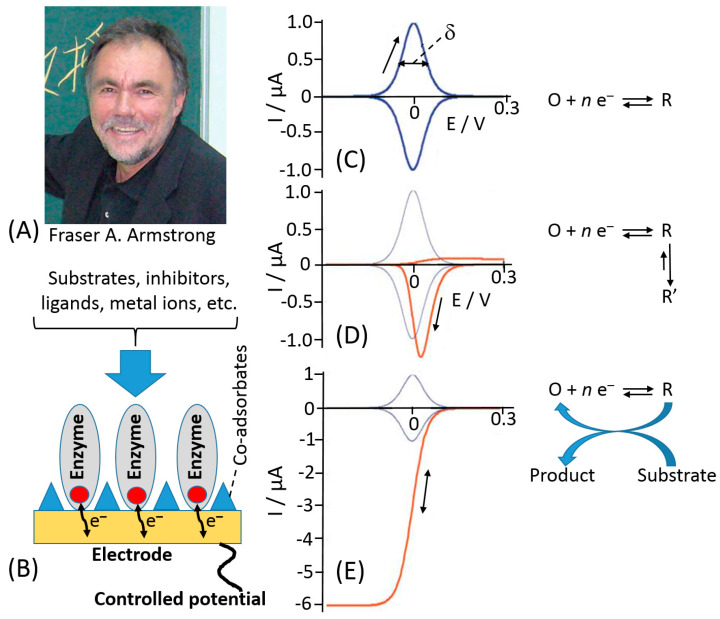
(**A**) Fraser A. Armstrong. (**B**) Cartoon showing an adsorbed monolayer of protein molecules on an electrode. Electron transfer accompanying a biocatalytic process is shown schematically. (**C**–**E**) Voltammograms expected for adsorbed redox couples displaying different types of ET coupling: (**C**) Reversible ET. (**D**) Orange trace shows ET coupled to a spontaneous chemical reaction of the reduced form; on this timescale, the reverse chemical process gates electron transfer. Blue trace shows uncoupled ET for comparison. (**E**) Orange trace shows ET coupled to catalytic regeneration of an oxidized form. Blue trace shows uncoupled ET. (The Armstrong photo is adopted from Wikipedia, public domain; Parts (**C**–**E**) were adopted from ref. [140] with permission.)

However, the main achievements were reported after introduction of a new investigation methodology reported by Fraser Armstrong (Figure 9A) as a protein-film voltammetry (PFV) [139,140,141]. In this technique, redox enzymes are “wired” directly to the electrochemical analyzer, which is able to activate and measure the redox behavior of the enzyme. A redox enzyme can be likened to an intriguing electronic device of which we would like to know all electronic features. To investigate its properties, we would plug the device to an electronic probe to measure all the parameters about its relays, switches, gates, etc., that have their representatives within the complex machinery of multi-centered redox enzymes as redox active sites [142]. The PFV provides a powerful way to investigate how ET processes occurs between active sites and electrodes and how the catalytic ET through an enzyme is controlled. The enzyme is adsorbed on a suitable electrode as a stable mono-/sub-monolayer film of molecules that are oriented to ease ET process [143], Figure 9B. This approach allows to overcome the problems of sluggish protein diffusion and kinetics limitations of the protein at the electrode. Therefore, PFV allows the detection and quantification of the complex and redox coupled chemical reactions that occur at the active sites [144].

Notably, the PFV exhibits several advantages in the investigation of the protein ET mechanism compared to other electrochemical techniques—(*i*) redox active centers are fully controlled through the electrode potential, thus allowing fine-tuning of the enzyme redox properties; (*ii*) well-defined curves especially considering the ideal case of a reversible ET mechanism that would give a couple of redox peaks with a Nerstian half-height width 90.6/*n* mV (where *n* is a number of electrons in the ET process) (Figure 9C)—by integrating the peak area, it is possible to determine the number of active sites within the protein layer; (*iii*) little amount of samples is needed to form a monolayer based on the assumption that the electrode surface can accommodate about 10^−12^–10^−11^ moles cm^−2^ of redox protein (other techniques require much bigger samples to get similar information); (*iv*) high sensitivity, considering the little amount of enzyme deposited onto the electrode; (*v*) high rate for ET reactions because they are not limited by diffusion. If the ET rate constant determined is at least 500 s^−1^ or higher, a chemical step with a very short half-life (milliseconds) can be coupled to the main ET reaction [145].

A completely different cyclic voltammogram from the one reported in Figure 9C can be obtained if an ET is followed by a spontaneous chemical process resulting in a product, which is reoxidized in a relatively slow reverse chemical process, showing an irreversible cyclic voltammogram, Figure 9D. Normally, the effect of catalytic turnover on the voltammetry depends primarily on how much mass transport of a substrate is limiting the reaction rate (current). This will be true in case of a macro-electrode coated with a high coverage of a very active enzyme. As the coverage or activity decreases, or if the electrode is a micro-electrode or one that is rotating at high speed, the current will more likely be determined by ET or properties of the enzyme. The catalytic turnover causes the peak-like signal to convert to a sigmoidal wave, where current is directly correlated to turnover rate, Figure 9E. Some of the well-studied examples include cytochrome P450, other heme-containing proteins, like hemoglobin, myoglobin, cytochrome c, and furthermore PS-I and PS-II photosystems, the proteins from the electron transfer chain, several hydrogenases, some Mo-containing proteins and various Fe–S, and other metal-containing proteins (mainly with Ni–Fe, Mn, or Cu as redox centers) [125,139,143].

In the last two decades, the PFV has been largely exploited to investigate the ET of many redox enzymes, that otherwise would remain unknown considering other techniques. Recently, the PFV has been coupled with special spectrophotometric techniques in order to monitor the variation in absorbance of a redox site while applying a specific potential at the electrode [146].

## 5. Different Approaches to Tackle DET Issues

Considering the advantages of the DET mechanism for the construction of many electrochemical biosensors, multiple different approaches to tackle the DET issues have been proposed over the past thirty years, like apo-enzyme reconstitution at an electrode surface, enzyme bioengineering, deglycosylation, site-oriented immobilization, and electrode nano-structuration [147,148,149,150,151,152,153,154].

Since the distance separating enzyme redox active catalytic centers and electrodes is the main problem for the DET, several approaches have been reported for decreasing this distance and facilitating the ET. One of the methods is based on plugging-in electronically conducting nanospecies, such as small Au nanoparticles or carbon nanotubes. This nano-size conducting bridges electronically connecting enzyme active centers and electrodes represent a nanotechnological approach to the electrical “wiring” of redox enzymes. It should be noted that the ultra-small size of the nano-bridges is critically important to allow their insertion into the protein globule for the efficient electrical contacting with the redox active centers located inside the protein.

**Figure 10:**
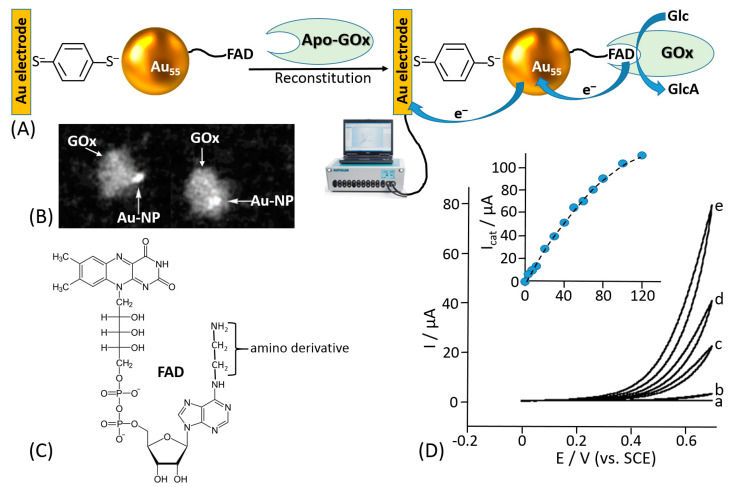
(**A**) Reconstitution of apo-GOx on FAD-functionalized Au nanoparticles operating as an electronically conducting nano-bridges facilitating electron transfer from the reduced FAD active center to the electrode support. (**B**) The scanning transmission electron microscopy (STEM) image showing binding of a single Au nanoparticle per the GOx enzyme molecule. (**C**) The synthetic FAD amino-derivative structure (note an additional amino group connected to the adenine with a spacer composed of two methylene groups). (**D**) Cyclic voltammograms corresponding to the bioelectrocatalyzed oxidation of glucose by the reconstituted GOx in the presence of different glucose concentrations: (a) 0 mM, (b) 1 mM, (c) 10 mM, (d) 20 mM, and (e) 50 mM. Results were recorded in 0.1 M phosphate buffer (pH 7.0), under Ar, potential scan rate 5 mV s^−1^. Inset: Calibration plot derived from the cyclic voltammograms at *E* = 0.6 V vs. SCE. (Part D was adopted from [155] with permission.)

**Figure 11:**
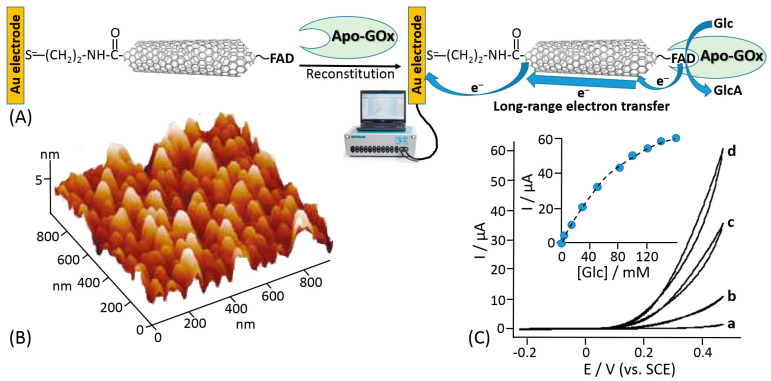
(**A**) Reconstitution of apo-GOx on FAD-functionalized carbon nanotubes operating as an electronically conducting nano-bridges facilitating long-range electron transfer from the reduced FAD active center to the electrode support. (**B**) Atomic force microscope (AFM) image of the GOx reconstituted on the FAD-functionalized carbon nanotubes monolayer associated with the Au electrode surface. (**C**) Cyclic voltammograms corresponding to the electrocatalyzed oxidation of different concentrations of glucose by the GOx reconstituted on the 25 nm-long FAD-functionalized carbon nanotube assembly: (a) 0, (b) 20, (c) 60 and (d) 160 mM glucose. Data recorded in phosphate buffer, 0.1 M, pH 7.4, scan rate 5 mV s^−1^. Inset: Calibration curve corresponding to the amperometric responses of the reconstituted GOx-electrode at E = 0.45 V in the presence of different concentrations of glucose. (Parts B and C were adopted from ref. [156] with permission.)

Willner et al. proposed reconstitution of an apo-flavoenzyme, namely apo-GOx, on a small gold nanocluster formed by 55 Au atoms (1.4 nm diameter) functionalized with the FAD cofactor [155] (Figure 10A). The gold nanoparticles were immobilized onto a gold electrode using a bifunctional thiol linker, benzene-1,4-dithiol, readily chemisorbed on the gold electrode producing a self-assembled monolayer, then attached to the Au nanoparticles with the second thiol group. The FAD derivative bound to the Au nanoparticles included an additional amino group linked to the cofactor unit through a short spacer (Figure 10C), which allowed the FAD cofactor to be kept separate from the nanoparticle to allow its reconstitution but still at a distance that provided the efficient electron transfer. In a control experiment, when the reconstitution of the apo-GOx with the FAD-functionalized Au nanoparticles was performed in a solution, scanning transmission electron microscopy (STEM) demonstrated a single Au nanoparticle bound to a GOx molecule (Figure 10B). Importantly, the reconstitution method resulted in the specific positioning of the Au nanoparticle near the active center of the reconstituted enzyme being partially embedded into the protein body, thus allowing efficient electron transfer from the FAD active center to the Au nanoparticle and then to the electrode support. Cyclic voltammetry measurements have demonstrated an electrocatalytic current corresponding to the glucose oxidation with the electron transfer through the Au nanoparticle operating as a conducting bridge (Figure 10D). This approach allowed obtaining an ET rate constant of approximately 5000 s^−1^. Despite the fact that the turnover number for the reconstituted enzyme was impressive and the ET was organized through the conducting bridge, the anodic electrocatalytic current was observed only with a very large overpotential starting at approximately +0.3 V (vs. SCE), while the FAD potential is ca. −0.45V (pH 7.0; SCE), thus requiring at least ca. 750 mV overpotential for the oxidation process (much bigger overpotential for the increased anodic current). A similar approach using carbon nanotubes functionalized with the same amino-FAD derivative for reconstituting apo-GOx has demonstrated an ET over a very long distance [156] (Figure 11). Both systems, where Au nanoparticles or carbon nanotubes have been used as conducting bridges, demonstrated quasi-direct ET transfer from the enzyme active centers to the electrode supports. The ET in these systems was not mediated by chemical redox species but rather provided by electronically conducting nano-wires. The major disadvantages of the reported systems [155,156] are the following: (*i*) a large overpotential originating from distances separating the nano-bridges and FAD cofactor and between the nano-bridges and the electrode surface and (*ii*) the use of the artificial (synthetic) FAD derivative (note that its synthesis is extremely complicated [148]). Both disadvantages resulted in low practical importance of the systems despite their scientific novelty. Particularly, the large overpotential for the anodic process did not allow use of these biocatalytic electrodes in biofuel cells.

**Figure 12:**
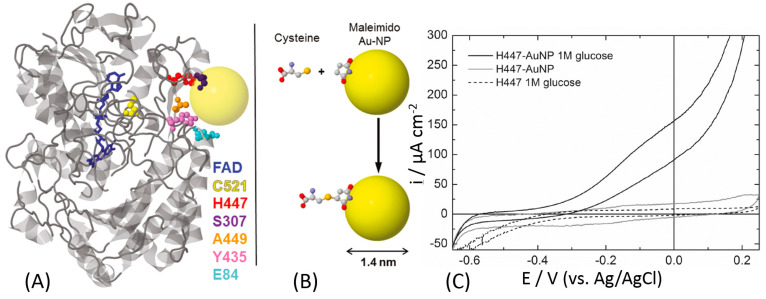
(**A**) Ribbon diagram of a GOx monomer (from *A. niger*) with the FAD molecule shown in blue. The amino acid residues targeted for mutagenesis are highlighted as space-filling models: cysteine (yellow), histidine (red), serine (purple), alanine (orange), tyrosine (pink), and glutamate (light blue). The yellow sphere represents an idealized Au nanoparticle (Au-NP) on the same scale as GOx. (**B**) Schematic drawing of the covalent-binding chemistry of cysteine to a maleimide-modified Au nanoparticle. The molecules are displayed as ball-and-stick: carbon (gray), oxygen (red), nitrogen (blue), and sulfur (yellow). (**C**) Cyclic voltammograms of H447C-Au-NP conjugates on a gold electrode in the presence (black line) and absence (gray line) of 1 M glucose (N_2_-saturated buffer, pH 7, 10 mV s^−1^). The cyclic voltammogram for unconjugated H44C is shown as a dotted line. The H447C-Au-NP conjugates in the presence of glucose exhibit enzymatic glucose oxidation starting at ca. −400 mV. The definition of all used mutants and their abbreviate names can be found in ref. [157]. (The figure was adopted from ref. [157] with permission.)

Banta and Atanassov et al. proposed a different approach to optimize the electrical communication between GOx and an electrode surface [157]. They introduced cysteine residues offering thiol groups into the GOx protein backbone by genetic engineering substituting natural amino acids, Figure 12A. Depending on the position of the newly introduced cysteine, the distance from its thiol group to the FAD cofactor was different ranging from 13.8 Å to 28.5 Å. The orientation of the GOx molecules at the electrode surface was controlled by the position of the artificially introduced cysteine used for the immobilization. Among all prepared GOx mutants, only H447C-mutant showed the DET activity as reported by the cyclic voltammetry that is displaying a small catalysis in the presence of glucose, in turnover conditions, Figure 12C. This might be ascribed to the low surface coverage of the enzyme. For this reason, the authors linked the mutated enzyme to a gold nanoparticle in order to facilitate the DET and increase the enzyme surface coverage. The artificially introduced thiol group was reacted with a maleimide-functionalized Au nanoparticles resulting in their covalent binding to the GOx protein backbone at the specific site, Figure 12B. Then, the Au-GOx mutant conjugate was bound to the electrode surface. The electrode modified with the H447C-Au nanoparticle showed a great catalytic current in the presence of the glucose substrate (Figure 12C). Unfortunately, the published cyclic voltammogram does not show clear redox peaks for FAD (in the absence of glucose, in non-turnover conditions), but the catalytic wave is starting at the potential close to the thermodynamic potential of FAD embedded in GOx, thus proving the DET features of the modified electrode. 

Recently, Gorton et al. proposed deglycosylation as a very innovative approach to tackle DET issues, especially based on their promising results previously obtained for horseradish and tobacco peroxidase, where the distance between the prosthetic group (*heme b*) and the electrode surface was effectively reduced, thus enhancing the DET rate. They proposed the same approach to enhance the ET rate of cellobiose dehydrogenase (CDH) [158]. In particular, they studied the effect of deglycosylation on two of most representative variants of CDH, namely CDH from *Phanerochaete chrysosporium* (*Pc*CDH) and *Ceriporiopsis subvermispora* (*Cs*CDH). Note that these enzymes are composed of two covalently linked domains, the catalytic dehydrogenase domain (CDH_DH_) and electron transfer cytochrome domain (CDH_Cyt_). The electron transfer proceeds as an internal interdomain process. Indeed, the study demonstrated that deglycosylation improves the catalytic current density, I_max_, and the sensitivity for lactose, as a substrate, which could be ascribed to a higher number of the electroactive CDH molecules at the electrode surface due to the downsizing of the enzyme’s dimensions and a facilitated DET due to the deglycosylation, which reduces the ET distance. Although the DET rate between CDH_CYT_ and the electrodes was increased, no DET between CDH_DH_ and the electrodes has been observed. The increased current density observed with the deglycosylated CDH-modified electrodes originates certainly from the decreased size of the deglycosylated CDHs. However, deglycosylation was also affecting the intrinsic kinetic parameters of the enzyme. The main drawbacks of this approach are the high cost and the impossibility to scale-up the process to industrial level for the production of very sensitive DET-based biosensors. However, the same approach has been used for pyranose dehydrogenase and other highly glycosylated enzymes [159,160,161].

Nowadays, one of the most used approaches to optimize DET rate or tackle DET issues is the site-oriented immobilization of redox enzymes through site-directed mutagenesis. Different specific protocols have been proposed by different research groups working in bioelectrochemistry. In order to obtain a productive orientation of the enzyme onto the electrode surface, it is needed a deep knowledge on the enzyme structure [162,163,164,165]. 

**Figure 13:**
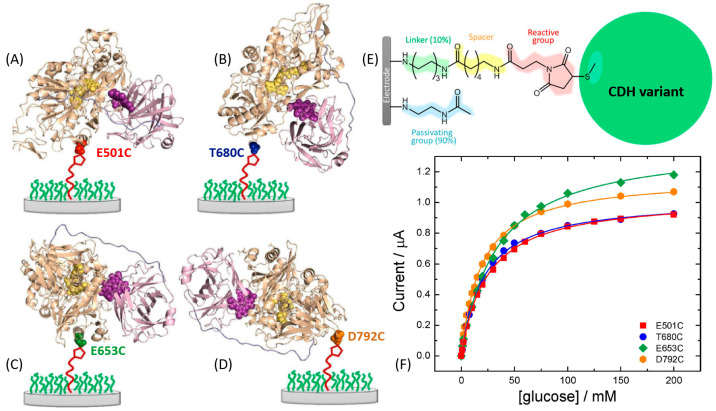
Cartoon representations of the structures of the four different *Mt*CDH variants attached to the electrode surface in different orientations through the cysteine-maleimide bond. The cytochrome domain is shown in purple and the dehydrogenase domain in pale brown. (**A**) E501C, substrate channel close to electrode (front on); (**B**) T680C, top of enzyme facing electrode (top on); (**C**) E653C, right side of substrate channel facing electrode (right side on); and (**D**) D792C, C-terminus close to electrode (bottom on). The images were obtained with PyMol software based on the crystal structure of *Mt*CDH, PDB code 4QI6. (**E**) Chemical structure of the whole electrode modification used in this work to immobilize *Mt*CDH variants with a single surface exposed cysteine, with the different components in different colors. (**F**) Background-corrected catalytic currents at 0.0 V vs. SCE for the four *Mt*CDH variants from parts (**A**–**D**) plotted as a function of the glucose concentration. The definition of all used mutants and their abbreviate names can be found in ref. [167]. (The figure was adopted from ref. [167] with permission.)

**Figure 14:**
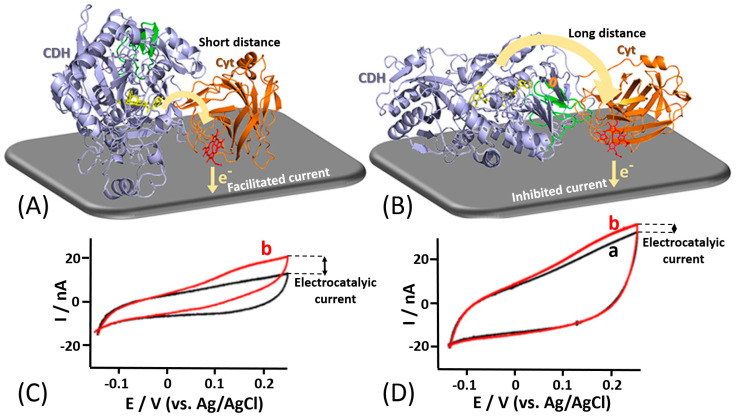
Direct electron transfer anisotropy of a site-specifically immobilized cellobiose dehydrogenase. (**A**) The enzyme immobilization with a short internal electron transfer path from a catalytic cellobiose dehydrogenase (CDH) domain to a cytochrome (Cyt) mediator domain connected with a flexible spacer. (**B**) The enzyme immobilization with a long electron transfer path. (**C**,**D**) Cyclic voltammograms obtained in the absence (a) and presence (b) of lactose (10 mM) for the enzyme immobilization with the short and long internal electron transfer distances, respectively. (The figure adopted from ref. [168] with permission.)

For example, Bartlett et al. reported on the covalent coupling between a surface-exposed cysteine residue and maleimide groups to immobilize different variants of *Myriococcum thermophilum* cellobiose dehydrogenase (*Mt*CDH) at multiwall carbon nanotube electrodes [166,167] (Figure 13E). By placing individual cysteine residues around the surface of the CDH_DH_ domain of the enzyme, they were able to immobilize the different variants with different orientations (Figure 13A–D). Notably, it was shown that DET occurs exclusively through the *heme b* cofactor and that the redox potential of the cofactor is unaffected by the orientation of the enzyme. This immobilization approach also resulted in an increased amount by 4–5 times of the electrically contacted (active) enzyme immobilized onto the electrode compared to not site-oriented immobilization. The current generated by the enzyme-modified electrodes in the presence of the cellobiose was dependent on the site-specific orientation of the enzyme-mutants (Figure 13F). In a similar approach [168], the same enzyme was immobilized onto a gold electrode by placing cysteine residues only around the CDH_DH_ domain in order to study the influence of CDH_CYT_ domain mobility on the ET rate (Figure 14A,B). For DET, the CDH_CYT_ domain needs to move from the closed-state conformation, where it obtains an electron from the catalytic CDH_DH_ to the open state where it can donate an electron to the electrode. Except for the optimal enzyme orientation (both domains on the side with the CDH_CYT_ in proximity of the electrode), CDH is not able to swing back the closed conformation, thus not allowing an efficient DET (Figure 14C,D). However, this approach does not necessarily require a site-directed mutagenesis as proposed in many papers. In some unique systems, a cysteine residue might be present at the optimum location near a redox active site already in natural structures, as it was the case for oriented immobilization of bacterial photosynthetic reaction centers at a modified electrode surface [169]. 

**Figure 15:**
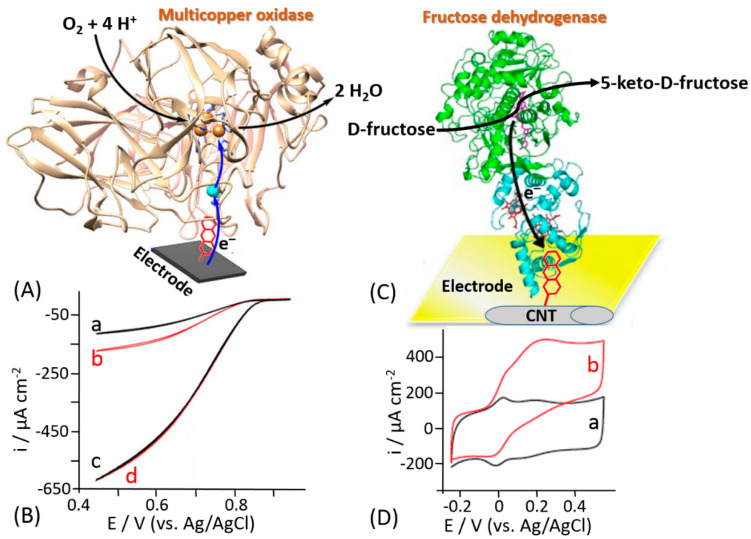
(**A**) Laccase (multicopper oxidase) site-specific immobilization due to the enzyme binding to the surface-located aromatic species. (**B**) The electrocatalytic activity of a film of *Pycnoporus cinnabarinus* laccase lcc3-1 (PcL) on an electrode without aromatic species and random orientation of the adsorbed enzyme (a–b) and on an electrode functionalized with the aromatic species providing orientation of the enzyme favorable for the DET (c–d). Cyclic voltammograms (a) and (c) correspond to the catalytic waves immediately after spotting on laccase solution. Cyclic voltammograms (b) and (d) show the catalytic waves after additional treatment of the modified electrode with a new portion of the enzyme. Potential scan rate was 5 mV s^−1^. (**C**) Fructose dehydrogenase site-specific immobilization due to the enzyme binding to the surface-located aromatic species. (**D**) Cyclic voltammograms measured with fructose dehydrogenase-electrode modified according the scheme shown in (**C**); in the absence (a) and presence of 10 mM D-fructose (b). The background electrolyte was 50 mM acetic buffer, pH 4.5; potential scan rate was 10 mV s^−1^. (Part A was adopted from [3] with permission; part B was adopted from [170] with permission; part D was adopted from [178] with permission.)

Different methods based on non-covalent enzyme binding have been applied for the oriented immobilization of enzymes in order to facilitate the DET. For example, Armstrong and his co-workers proposed the immobilization of laccase through a hydrophobic pocket nearby one of the metal centers included in the enzyme, namely T1 copper (T1Cu), in order to enhance the DET rate [170]. Notably, laccases belong to the family of multicopper oxidases (MCOs), where the ET proceeds through the following three steps: (*i*) the reduction of the T1Cu site through the electrons transferred from a substrate (or electrode considering the immobilized enzyme), (*ii*) the internal electron transfer (IET) or tunneling between the T1Cu and trinuclear copper cluster (TNC) proceeding through the Cys-(His)_2_ bridge over a distance of 13 Å, and (*iii*) O_2_ reduction taking place at TNC [171,172]. The authors proposed the electrodeposition of diazonium salts of a wide group of aryl amines, thus producing a highly aromatic electrode surface that would be able to access the hydrophobic pocket of the enzyme (Figure 15A). The cyclic voltammogram for an unmodified carbon electrode with randomly adsorbed laccase showed a very small catalytic wave (Figure 15B, curve a) that was doubled upon electrode incubation with additional amount of the enzyme (Figure 15B, curve b), thus showing a partial coverage of the electrode. On the other hand, using the site-oriented immobilization the DET rate was greatly improved by at least six-fold (Figure 15B, curve c.) Upon further incubation with an additional amount of enzyme, an overlapping cyclic voltammogram was recorded (Figure 15B, curve d), meaning that a full enzyme coverage was achieved. This approach to the enzyme immobilization has been further used mainly for the biofuel cell development because of the minimization of overpotential needed to activate the reduction of O_2_ at the cathode surface [173,174,175,176,177]. 

The same approach has been reported by Bollella et al. for the immobilization of a carbohydrate oxidizing enzyme, namely fructose dehydrogenase (FDH) [178]. An efficient DET reaction pathway between FDH and a carbon nanotube-modified electrode further grafted with an aromatic compound has been reported (Figure 15C). Anthracene molecules have been deposited onto single walled carbon nanotubes (SWCNTs) by electrochemical reduction of 2-aminoanthracene diazonium. Cyclic voltammograms measured in the absence of D-fructose with the FDH-modified electrode revealed two couples of redox waves attributed to *heme c_1_* and *heme c_3_* of the cytochrome domain (Figure 15D, curve a). The addition of 10 mM D-fructose, which is a substrate of FDH, resulted in two catalytic waves correlated with *heme c_1_* and *heme c_3_* with a maximum current density of 485 ± 21 μA cm^−2^ (Figure 15D, curve b). Conversely, only one couple of redox peaks and one catalytic wave in the absence and presence of D-fructose, respectively, were observed for the plain carbon nanotube-modified electrode. The difference has been explained by different orientation of the FDH enzyme molecules at the electrode surface. Indeed, the FDH molecules are randomly adsorbed at the electrode surface in the absence of the anthracene grafted at SWCNTS. On the other hand, the hydrophobic pocket close to the *heme* groups of the cytochrome domain interact with the grafted anthracene due to the π-π interactions with the aromatic side chains of the amino acids present in the hydrophobic pocket of FDH [179,180]. This interaction results in the oriented deposition of the FDH molecules facilitating redox transformations for both *heme* groups according to the DET mechanism. 

Although the discussed approaches allowed to enhance the DET rate for many enzymes, they exhibit as main drawbacks the high cost and complexity of the modified electrode preparation, which do not allow them to scale-up to an industrial level. Therefore, the efficient ET between enzymes and electrodes is still recognized by the scientific community as the major challenge. Nanotechnological approaches facilitating the ET, also increasing the enzyme load, remain as active research directions [27,110,181,182]. Among all kinds of electrode materials, metal nanoparticles and carbon-based nanomaterials play an important role in the electrode modification, because of their high surface area-to-volume ratios and high surface energy, which facilitate immobilization of enzymes, allowing them to act as electron conducting pathways between the prosthetic groups of the enzymes and electrode surfaces. Due to the high research activity in this area, the advances in this research direction have been extensively reviewed [183,184,185]. 

## 6. Conclusions and Future Perspectives

The investigation of the electrochemical properties of biological materials has gained a solid foundation over the past 80 years. From the very first observation of the protein electrochemical activity in polarographic measurements reported by Brdička in 1930s [186,187,188], great achievements [189] have been progressed in electrochemical studies of redox proteins, enzymes, and whole biological cells (e.g., bacterial cells, yeast cells, etc.). The success in bioelectrochemistry has been achieved by using carefully designed chemically modified electrode surfaces and particular experimental conditions [51]. Recently, many bioelectrochemists successfully attempted to tackle ET issues for various redox enzymes with detailed understanding of the ET mechanism. Despite the general progress on the ET mechanism elucidation for many redox enzymes, the ET mechanism of GOx still remains an open debate based on experimental evidences reported in the literature from both sides (pro and quo). On the one hand, many researchers are claiming that GOx immobilized on an electrode surface cannot undergo DET reaction mechanism, without considering the reliability and the “correct” scientific meaning or interpretation of the results. On the other hand, other scientists are claiming the absence of FAD dissociation from the enzyme structure, thus suggesting glucose can be determined directly either by the redox process of the co-enzyme FAD or by oxygen consumption (competitive mechanism) [190,191,192,193].

In the future, the development of all these technologies will be certainly scaled-up to the industrial level allowing to take great advantages of fundamental studies in bioelectrochemistry [194,195]. The future developments in bioelectrochemistry are related to understanding the ET mechanism of more complex biological species (e.g., whole cells, etc.), possibly extending the numbers of enzymes that could be connected in DET to an electrode surface. However, bioelectrochemistry is also connected to the developments in different scientific and technological areas, like bioengineering and materials science. The former is mainly important for the biological mutation of redox enzymes, while the latter is important for the synthesis or electrosynthesis of new nano-catalysts used to support the DET of many redox enzymes [196]. Although the analytical aspects of the ET mechanisms have not been herein reviewed, as the development of biosensors, we summarized all the fundamentals needed for newcomers in order to enrich their knowledge about bioelectrochemistry [197,198]. Besides biosensing applications [199,200], bioelectrochemisty can also find applications for the development of special power-generating systems (e.g., enzyme-based [201,202,203,204,205,206] and microbial [207,208,209] biofuel cells) or employed as a biocomponent in unconventional information processing systems [210,211].
